# Atrial fibrillation and risk of myocardial infarction by sex and time since diagnosis. The Tromsø Study 1994–2021

**DOI:** 10.1093/ehjqcco/qcag039

**Published:** 2026-04-24

**Authors:** Linn Nilsen, Ekaterina Sharashova, Maja-Lisa Løchen, Jan T Mannsverk, Tom Wilsgaard

**Affiliations:** Department of Community Medicine, UiT The Arctic University of Norway, Hansine Hansens veg 18, 9012 Tromsø, Norway; Department of Community Medicine, UiT The Arctic University of Norway, Hansine Hansens veg 18, 9012 Tromsø, Norway; Department of Community Medicine, UiT The Arctic University of Norway, Hansine Hansens veg 18, 9012 Tromsø, Norway; Department of Clinical Medicine, UiT The Arctic University of Norway, 9012 Tromsø, Norway; Department of Cardiology, University Hospital of North Norway, 9012 Tromsø, Norway; Department of Clinical Medicine, UiT The Arctic University of Norway, 9012 Tromsø, Norway; Department of Cardiology, University Hospital of North Norway, 9012 Tromsø, Norway; Department of Community Medicine, UiT The Arctic University of Norway, Hansine Hansens veg 18, 9012 Tromsø, Norway

**Keywords:** Atrial fibrillation, Myocardial infarction, ST-segment elevation myocardial infarction, Non-ST-segment elevation myocardial infarction, Atrial fibrillation management, Sex differences

## Abstract

**Aims:**

The relationship between atrial fibrillation (AF) and myocardial infarction (MI) remain unclear. We aimed to examine the sex-specific risk of MI, ST-segment elevation MI (STEMI) and non-ST-segment elevation MI (NSTEMI) in participants with AF, overall and by time since AF diagnosis.

**Methods and results:**

We followed 25 132 participants (53% women) from the Tromsø Study (1994–95) without AF and MI at baseline through 2021. Cox proportional- and Fine-Gray sub-distribution-hazard regression assessed AF as a risk factor for MI, STEMI and NSTEMI overall and by time since AF diagnosis (0 to <0.5, 0.5 to <1, 1 to <5, 5 to <10 and ≥10 years). During follow-up, 115 women and 124 men experienced incident AF and subsequent MI. AF was associated with increased hazard of MI in women (sub-distribution hazard ratio [sHR] 1.39, 95% confidence interval [CI] 1.12–1.72), but not in men. In women, the hazard of MI increased 1–10 + years since AF, peaking at ≥10 years (sHR 1.75, 95% CI 1.07–2.84). In men, hazard was increased within the first six months (sHR 2.27, 95% CI 1.45–3.54) but not thereafter. STEMI hazard was increased only in men within six months. NSTEMI hazard was elevated in women 1–10 + years since AF and in men within the first year.

**Conclusion:**

AF is a risk factor for MI in both sexes, but risk patterns differ. Women are at increased hazard of NSTEMI 1–10 + years since AF, while men are at increased hazard of STEMI within six months and NSTEMI within the first year.

## Structured graphical abstract

**Structured graphical abstract:**
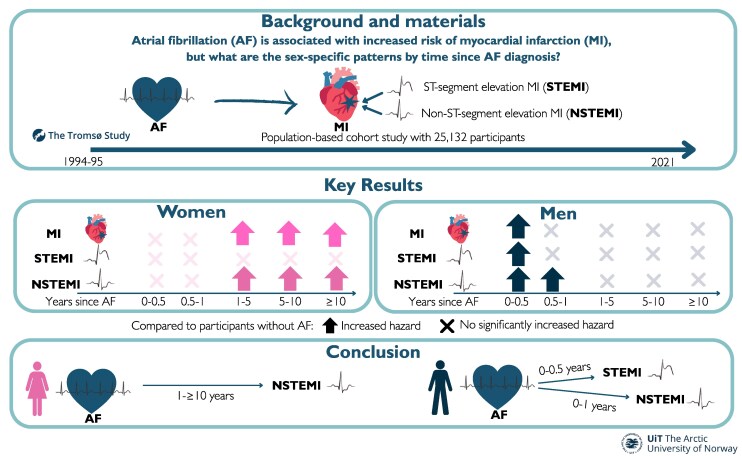

Key Learning Points What is already known

- AF and MI are risk factors for each other
- Disease onset of AF and MI seem to cluster within 30 days from each other

What this study adds

- Risk pattern for MI after AF differ between women and men
- Women are at increased risk of NSTEMI after the first years since AF, and onwards
- Men are at increased risk of STEMI within the first six months after AF, and of NSTEMI within the first year after AF. After the first year since AF, no significantly increased hazard of MI was found in men.

## Introduction

Atrial fibrillation (AF) and myocardial infarction (MI) impose public health challenges worldwide, together accounting for a large part of total cardiovascular disease burden.^1^ They share risk factors like age, obesity and hypertension and are risk factors for each other.^2,3^ A systematic review from 2017 found that AF was associated with a 54% higher risk of MI [95% confidence interval (CI) 1.28–1.85].^3^ The risk of MI was higher in women with AF than in men.^4,5^ The combination of AF and MI is associated with a two times higher hazard of mortality, compared to MI without AF.^5^

The pathophysiological interactions between AF and MI are not fully understood. However, systemic inflammation, endothelial dysfunction, tachycardia and coronary thromboembolism are highlighted as possible explanations.^6^ Understanding whether AF is a risk factor for ST-segment elevation MI (STEMI) and/or non-ST-segment elevation MI (NSTEMI) could enhance our understanding of the association between AF and MI. To our knowledge, only two studies have investigated the association between AF and risk of MI by type (STEMI vs. NSTEMI), and no study has performed this sex-specific analysis. Soliman *et al.* found no association with STEMI but found that AF was associated with an 80% increased hazard of NSTEMI.^4^ Conversely, Frederiksen *et al.* reported that AF was associated with 84% increased hazard of STEMI and a 2.70 times higher hazard of NSTEMI.^5^ Thus, current literature is limited and inconclusive. Additionally, even though both studies found a higher relative risk of overall MI in women with AF than in men, they adjusted for sex rather than stratified in their sub-analyses of risk of MI by type.^4,5^ There is therefore a need to confirm if the sex difference in risk of MI among AF-patients is present for STEMI and/or NSTEMI.

Moreover, the role of temporality in the association between AF and MI is unclear. Studies have found a clustering of disease onset of AF and MI within 30 days of each other,^7–10^ but no study has investigated the sex-specific risk of MI in AF-patients by time since first AF-diagnosis. More knowledge about this temporal relationship could further enhance our understanding of the interplay between AF and MI and can have clinical implications.

We used a large, population-based cohort study from Norway to examine the risk of MI, STEMI and NSTEMI in women and men with AF at any time, and by time since first diagnosis of AF.

## Methods

### Study population

**Figure 1:**
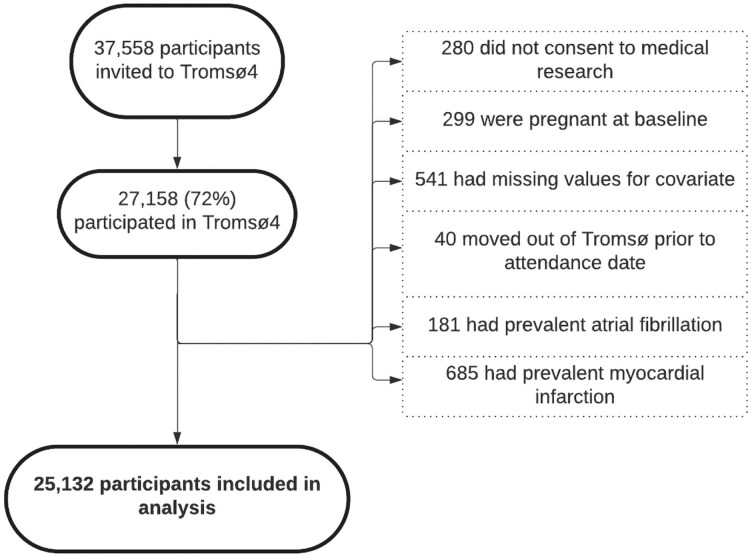
Flow chart of study participants. The Tromsø Study 1994–2021.

Our sample included 25 132 participants from the fourth wave of the Tromsø Study (Tromsø4, 1994–95), followed up for incident AF and MI until 31 December 2021. The Tromsø Study is a large, ongoing, population-based cohort study conducted in seven completed waves in the municipality of Tromsø, Northern Norway, since 1974.^11^ In Tromsø4, all residents aged 25 years or older were invited to participate, and *n* = 27 158 (75% of invited women and 70% of invited men) participated.^11^ We excluded participants who did not consent to medical research (*n* = 280), those who moved out of the municipality prior to their examination date (*n* = 40), participants with incomplete covariate history (*n* = 541) and participants with prevalent AF (*n* = 181) or MI (*n* = 685) or who were pregnant (*n* = 299) at baseline (*Figure 1*). All participants gave a written informed consent. The Tromsø Study complies with the 1964 Declaration of Helsinki and its later amendments and is approved by the Regional Committee for Medical and Health Research Ethics, North Norway, and by the Norwegian Data Protection Authority.

### Data collection

Before the examination, participants were invited to fill in a four-page questionnaire to be handed in at the examination. Additionally, a more comprehensive questionnaire was handed out at the physical examination, to be filled in at home and then delivered by mail in a prepaid envelope. The physical health examination included, among others, measurement of height, weight and blood pressure, and blood sample collection, and is described in detail elsewhere.^11^ Information collected from questionnaires included use of hypertensive medications (current user; previous user; never user), history of diabetes (no/yes), hours of hard physical activity per week (none; less than 1; 1–2; 3 or more), daily cigarette, cigar or pipe smoking (no/yes to each of three questions) and education [7–10 years primary/secondary school; technical school, middle school, vocational school, 1–2 years senior high school; high school diploma (3–4 years); college/university, less than 4 years; college/university, 4 or more years]. Information collected at the physical examination included weight, height, systolic and diastolic blood pressure. Weight and height were measured without shoes and with light clothes, and body mass index (BMI) was calculated as weight (kg) divided by height (m) squared. Blood pressure was measured three times with one-minute intervals after two minutes of rest, using Dinamap Vital Signs Monitor 1846 (Critikon Inc., Tampa, FL, USA), and the mean of the last two measures was used. Additionally, non-fasting blood samples collected at the physical examination were analysed for total cholesterol and high-density lipoprotein cholesterol (HDL). We combined the variables for systolic and diastolic blood pressure and use of hypertensive medications to create a variable for hypertension control (normotensive (SBP <140 mmHg, DBP <90 mmHg *and* not using antihypertensive medications); controlled hypertension (SBP <140 mmHg, DBP <90 mmHg *and* using antihypertensive medications); uncontrolled hypertension (SBP ≥140 mmHg or DBP ≥90 mmHg *and* using antihypertensive medications); untreated hypertension (SBP ≥140 mmHg or DBP ≥90 mmHg *and* not using antihypertensive medications).

### Identification and follow-up of incident atrial fibrillation and myocardial infarction

All participants were followed up from the date of participation in Tromsø4 in 1994–95 until whichever came first of incident MI, emigration, death or end of follow-up on 31 December 2021. This was done by linkage to the local diagnosis registry at the University Hospital of North Norway (UNN), the Norwegian Myocardial Infarction Register, the Population Registry of Norway and the Norwegian Cause of Death Registry using the unique 11-digit Norwegian personal identification number.

Incident cases of AF were identified until 31 December 2016 through the local registry at UNN. In the local registry, potential cases of AF were selected for validation through a broad search using International Classification of Diseases (ICD) codes (ICD-9 codes 410–414, 427, 428, 430–438, and 798–799, and ICD-10 codes I20-I25, I46-I48, I50, I60-I69, R96, R98 and R99.^12^) Additionally, hospital records were searched manually for notes on AF in patients with cardiovascular or cerebrovascular events. All cases were validated by an independent endpoint committee, and AF were confirmed by electrocardiogram (ECG). Participants without confirmation of AF by ECG (*n* = 141) and with AF during the last 7 days of life (*n* = 40) were considered AF-free in the analysis. The local AF-registry at the local hospital UNN, is updated and validated through 2016 only. Therefore, from 1 January 2017 until 31 December 2021, incident cases of AF were identified through discharge records of AF from UNN.

Incident cases of MI were identified until 31 December 2014 through the local registry at UNN. In the local registry, potential cases of MI were selected for validation by searching the discharge registry for ICD-8 codes 410–414, 427, 795–796 in the period 1960–1979, ICD-9 codes 410–414, 427.5, 798 and 799 in the period 1980–98, and thereafter ICD-10 codes I20–I25, I46-I48, I50, R96, R98 and R99,^13^ Additionally, hospital records were searched manually for notes on MI in patients with cerebrovascular events, transient cerebral ischaemic attacks, vascular syndromes of the brain and hemiplegia and hemiparesis (ICD-10 coded I60-I69, G45, G46 or G81). All cases of MI were reviewed and validated by a local endpoint committee, using Modified World Health Organisation–MORGAM (MONICA Risk, Genetics, Archiving and Monograph) criteria, based on clinical symptoms and findings, findings in ECGs, values of cardiac biomarkers, and autopsy reports when applicable. The local MI-registry at UNN is updated and validated through 2014 only. Therefore, from 1 January 2015, incident cases of MI were identified through the Norwegian Myocardial Infarction Register. This registry has been described in detail elsewhere and has been found to be highly correct and sufficiently complete in order to be used as an endpoint source for population-based epidemiological studies.^14,15^ Fatal cases of MI out of hospital were identified by linkage to the National Cause of Death Registry through ICD-10 codes I20-I24, I25.2, I46, or R96 combined with I20 or I25.

From the local registry, MI cases were classified as STEMI using criteria from the First Universal Definition of MI.^16^ Specifically, MI-cases were classified as STEMI if significant ST-elevation (≥2 mm in V1, V2 and V3 and ≥1 mm in the other leads) was observed in ECG at admission in ≥2 contiguous leads. If a patient was classified as having MI, but information on ST-elevation was missing (*n* = 494), the case was classified as STEMI if information from ECG indicated 1) development of new, significant pathological Q-wave > 3 mm wide or ≥2 mm in V2-3 and amplitude ≥25% of subsequent R-wave amplitude, 2) clear, pathological Q-wave present at admission and development of ST-T change and/or development of persistent negative T-wave, or 3) clear new Q-wave described in journal, or other clear indications of Q-wave myocardial infarctions (*n* = 21). From the national registry, cases were classified as STEMI if significant ST-elevation (new ST-elevation at the J-point in two contiguous leads with the cut-point ≥ 1 mm, except for leads V2-3, where it should be ≥2 mm in men ≥40 years, ≥2.5 mm in men < 40 years, and ≥1.5 mm in women) was observed in the ECG.^17^ From both the local and the national registry, cases not classified as STEMI were classified as NSTEMI if no indications of left bundle branch block were found and if information on ST-elevation and ECG was not missing. Cases not classified as STEMI, NSTEMI or left bundle branch block and with uncertain ECG or with missing information on ST-elevation were classified as unclassified MI.

### Statistical analysis

All analyses were performed sex-specifically using SAS software version 9.4 (SAS Institute, Cary, NC, USA). Descriptive characteristics of the sample were presented by means, standard deviations (SD), frequencies and proportions. We also explored descriptive characteristics of the participants stratified by AF and/or MI-status.

The hazard of incident MI (all subtypes), NSTEMI and STEMI after AF was examined using Cox proportional hazard regression and Fine-Grey subdistribution hazard regression.^18^ We used three models, whereas model 1 was adjusted for age only, model 2 additionally was adjusted for BMI, total cholesterol, HDL, hypertension control, history of diabetes, hours of hard physical activity per week, daily smoking and educational level, and model 3 additionally had non-MI death, NSTEMI, STEMI, left bundle branch block, unclassified MI and fatal MI treated as competing risks (non-MI death as competing risk in analysis for total MI; non-MI death, STEMI, left bundle branch block, unclassified MI and fatal MI as competing risk in analysis for NSTEMI; non-MI death, NSTEMI, left bundle branch block, unclassified MI and fatal MI as competing risk in analysis for STEMI). Thus, models 1 and 2 present hazard ratios (HRs) and model 3 presents subdistribution hazard ratios (sHRs). AF was treated as a time-varying covariate, and attained age was used as the timescale. To examine the risk of MI, NSTEMI and STEMI by time since first diagnosis of AF, the SAS Lexis macro was used to create breaks at selected time intervals (0 to <0.5 years, 0.5 to <1 years, 1 to <5 years, 5 to <10 years and ≥10 years) since the date of AF. The proportional hazards assumption for each time period since AF diagnosis was checked by the Schoenfeld residuals test. Forest plots were created for sHRs from model 3.

Cumulative incidence curves were produced from Cox models, corresponding to model 3 described above, and presented by time period since AF diagnosis.

As a sensitivity analysis, we repeated the sub-distribution hazard regression of model 3 with cases of fatal MI, left bundle branch block, and unclassified MI included as cases of STEMI. This was done in three models, whereas model 1 included cases with fatal MI as STEMI cases, model 2 included fatal cases *and* cases with left bundle branch block, and model 3 additionally included cases with unclassified MI.

## Results

### Baseline characteristics

We followed 13 299 women and 11 903 men for a median follow-up time of 20.2 years (*Table 1*). The mean age was 46.9 years in women and 45.7 years in men. Mean (SD) BMI was 24.7 (4.2) kg/m^2^ in women and 25.6 (3.3) kg/m^2^ in men. Mean (SD) total cholesterol was 6.0 (1.4) mmol/L in women and 6.0 (1.2) mmol/L in men and mean (SD) HDL was 1.6 (0.4) mmol/L in women and 1.4 (0.4) mmol/L in men. A total of 69% of women and 60% of men were normotensive, whereas 24% of women and 35% of men had untreated hypertension. The history of diabetes was prevalent in 1.6% of women and 1.5% of men. In women, 75.6% were physically inactive (active for less than one or no hours per week), 37% were daily smokers, and 28% had a university/college education. In men, 62% were physically inactive, 38% were daily smokers, and 32% hada university/college education. Characteristics of participants by AF and/or MI-status are presented in Supplementary material online, *Table S1*. During follow-up, in women and men, respectively, we observed 967 and 1058 incident cases of AF, 1000 and 1611 incident cases of MI, and 115 and 124 incident cases of AF *and* subsequent MI (*Table 2*).

**Table 1:** Descriptive characteristics by sex^a^

	Women	Men
N	13 229	11 903
Age, years	46.9 (15.19)	45.7 (14.04)
Body mass index, kg/m^2^	24.7 (4.24)	25.6 (3.29)
Total serum cholesterol, mmol/L	6.03 (1.38)	6.02 (1.22)
High-density lipoprotein cholesterol, mmol/L	1.64 (0.40)	1.35 (0.35)
Hypertension control		
Normotensive	9172 (69.3)	7122 (59.8)
Controlled hypertension	163 (1.2)	146 (1.2)
Uncontrolled hypertension	698 (5.3)	499 (4.2)
Untreated hypertension	3196 (24.2)	4136 (34.7)
History of diabetes	209 (1.6)	178 (1.5)
Hours of hard physical activity per week		
None	7422 (56.1)	4698 (39.5)
Less than 1	2579 (19.5)	2652 (22.3)
1–2	2378 (18.0)	2745 (23.1)
3 or more	850 (6.4)	1808 (15.2)
Daily smoker	4904 (37.1)	4510 (37.9)
Education		
Primary education	4942 (37.4)	3555 (29.9)
Upper secondary education	4573 (34.6)	4499 (37.8)
University/college <4 years	1851 (14.0)	1947 (16.4)
University/college ≥4 years	1863 (14.1)	1902 (16.0)

The Tromsø Study 1994–2021.

^a^Numbers are given as mean (standard deviation) or number (percentage).

**Table 2:** Incident atrial fibrillation and myocardial infarction during follow-up

	Women	Men
Incident AF during follow-up	967 (7.3)	1058 (8.9)
Incident MI during follow-up	1000 (9.4)	1611 (16.6)
STEMI	239 (23.9)	500 (31.0)
NSTEMI	512 (51.2)	788 (48.9)
Left bundle branch block	40 (4.0)	50 (3.1)
Fatal MI	43 (4.3)	55 (3.4)
Unclassified MI	166 (16.6)	218 (13.5)
Incident AF *and* subsequent MI during follow-up	115 (0.9)	124 (1.1)
STEMI	10 (8.7)	18 (14.5)
NSTEMI	72 (62.6)	62 (50.0)
Left bundle branch block	5 (4.3)	9 (7.3)
Fatal MI	6 (5.2)	21 (16.9)
Unclassified MI	22 (19.1)	14 (11.3)

The Tromsø Study 1994–2021.

Percentages for incident AF, MI and AF *and* subsequent MI during follow up is for the proportion of the total sample. Percentages for subtypes of MI are for the proportion of all cases.

AF, Atrial fibrillation; MI, Myocardial infarction; NSTEMI, non-ST-segment elevation myocardial infarction; STEMI, ST-segment elevation myocardial infarction.

### Atrial fibrillation and risk of incident myocardial infarction

HRs and sHRs of MI, STEMI and NSTEMI after AF at any time are presented in *Table 3*. In the fully adjusted model without competing risks considered (model 2), women with AF at any time had 58% (95% CI 29%, 94%) higher hazard of MI, no increased hazard of STEMI and 87% (44%, 143%) higher hazard of NSTEMI, as compared with women without AF. In the model with competing risks considered (model 3), sHRs were 1.39 (1.12, 1.72) for MI, 0.66 (0.34, 1.28) for STEMI and 1.78 (1.35, 2.35) for NSTEMI. In men, a significant HR was found only for MI after AF at any time in the fully adjusted model without competing risks considered (model 2) (HR 1.25, 95% CI 1.03, 1.51).

**Table 3:** Hazard ratios for incident MI, STEMI and NSTEMI according to AF at any time or time since first diagnosis of AF

	Women			Men		
		Hazard ratio (95% confidence interval)^d^				
	Model 1^a^	Model 2^b^	Model 3^c,d^	Model 1^a^	Model 2^b^	Model 3^b,d^
	**MI**					
No AF	**1 (Reference)**	**1 (Reference)**	**1 (Reference)**	**1 (Reference)**	**1 (Reference)**	**1 (Reference)**
Time since AF						
0 to <0.5 years	1.13 (0.51, 2.53)	1.09 (0.49, 2.44)	0.64 (0.28, 1.46)	3.07 (2.01, 4.69)	3.06 (2.00, 4.67)	2.27 (1.45, 3.54)
0.5 to <1 year	1.22 (0.55, 2.73)	1.16 (0.52, 2.60)	0.92 (0.41, 2.10)	1.66 (0.91, 3.01)	1.67 (0.92, 3.03)	1.54 (0.84, 2.82)
1 to <5 years	1.48 (1.10, 2.00)	1.46 (1.08, 1.98)	1.37 (1.00, 1.87)	1.18 (0.88, 1.58)	1.20 (0.89, 1.61)	1.14 (0.85, 1.54)
5 to <10 years	1.98 (1.42, 2.76)	1.93 (1.38, 2.69)	1.72 (1.21, 2.44)	0.93 (0.63, 1.39)	0.93 (0.62, 1.38)	0.94 (0.63, 1.42)
≥10 years	1.97 (1.24, 3.13)	1.80 (1.13, 2.87)	1.75 (1.07, 2.84)	0.93 (0.59, 1.47)	0.96 (0.60, 1.51)	0.96 (0.60, 1.53)
AF at any time	1.64 (1.34, 2.00)	1.58 (1.29, 1.94)	1.39 (1.12, 1.72)	1.23 (1.019, 1.49)	1.25 (1.03, 1.51)	1.19 (0.98 1.45)
	**STEMI**					
No AF	**1 (Reference)**	**1 (Reference)**	**1 (Reference)**	**1 (Reference)**	**1 (Reference)**	**1 (Reference)**
Time since AF						
0 to <0.5 years	NA	NA	NA	2.73 (1.13, 6.62)	2.75 (1.13, 6.69)	2.08 (0.85, 5.10)
0.5 to <1 year	NA	NA	NA	NA	NA	NA
1 to <5 years	1.03 (0.46, 2.35)	0.96 (0.42, 2.19)	0.93 (0.40, 2.14)	0.68 (0.32, 1.45)	0.70 (0.33, 1.49)	0.74 (0.35, 1.59)
5 to <10 years	0.60 (0.15, 2.42)	0.55 (0.13, 2.22)	0.51 (0.12, 2.13)	0.44 (0.41, 1.38)	0.45 (0.15, 1.42)	0.52 (0.16, 1.64)
≥10 years	1.22 (0.30, 4.98)	1.037 (0.25, 4.24)	1.12 (0.28, 4.54)	0.60 (0.19, 1.87)	0.65 (0.21, 2.056)	0.75 (0.24, 2.36)
AF at any time	0.78 (0.41, 1.49)	0.71 (0.37, 1.36)	0.66 (0.34, 1.28)	0.71 (0.44, 1.14)	0.73 (0.45, 1.19)	0.77 (0.47, 1.26)
	**NSTEMI**					
No AF	**1 (Reference)**	**1 (Reference)**	**1 (Reference)**	**1 (Reference)**	**1 (Reference)**	**1 (Reference)**
Time since AF						
0 to <0.5 years	1.47 (0.55, 3.94)	1.45 (0.54, 3.90)	0.91 (0.33, 2.49)	3.12 (1.71, 5.69)	3.06 (1.68, 5.58)	2.14 (1.15, 4.01)
0.5 to <1 year	1.15 (0.37, 3.58)	1.12 (0.36, 3.49)	0.96 (0.30, 3.026)	2.38 (1.18, 4.80)	2.33 (1.16, 4.70)	2.27 (1.11, 4.62)
1 to <5 years	1.59 (1.07, 2.38)	1.60 (1.07, 2.40)	1.60 (1.06, 2.41)	0.94 (0.59, 1.48)	0.93 (0.59, 1.48)	0.92 (0.58, 1.47)
5 to <10 years	2.41 (1.60, 3.64)	2.38 (1.57, 3.59)	2.33 (1.52, 3.58)	1.00 (0.59, 1.71)	0.97 (0.57, 1.65)	1.06 (0.62, 1.82)
≥10 years	2.42 (1.41, 4.17)	2.24 (1.30, 3.87)	2.48 (1.40, 4.37)	0.89 (0.48, 1.68)	0.89 (0.47, 1.67)	0.94 (0.49, 1.79)
AF at any time	1.90 (1.46, 2.46)	1.87 (1.44, 2.43)	1.78 (1.35, 2.35)	1.19 (0.91, 1.56)	1.17 (0.89, 1.54)	1.17 (0.88, 1.55)

The Tromsø Study 1994–2021.

AF, atrial fibrillation; MI, Myocardial infarction; NSTEMI, non-ST-segment elevation myocardial infarction; STEMI, ST-segment elevation myocardial infarction.

^a^Adjusted for age.

^b^Model 1 + adjusted for body mass index, total cholesterol, high-density lipoprotein cholesterol, hypertension control (normotensive/controlled hypertension/uncontrolled hypertension/untreated hypertension), diabetes, hours of hard physical activity per week, current daily smoking and education level as additional covariates.

^c^Model 2 + death, STEMI, NSTEMI, left bundle branch block, unclassified MI and fatal MI treated as competing risks (death as competing risk in analysis for total MI; death, STEMI, left bundle branch block, unclassified MI and fatal MI as competing risk in analysis for NSTEMI; death, NSTEMI, left bundle branch block, unclassified MI and fatal MI as competing risk in analysis for STEMI).

^d^Estimates for model 3 are subdistribution hazard ratios.

### Temporal relationship between atrial fibrillation and incident myocardial infarction

Women with AF had no significantly increased hazard of MI during the first year since AF diagnosis, as compared to women without AF (*Table 3*). However, at 1 to <5 years, 5 to <10 years and ≥10 years, sHRs of MI were 1.37 (1.00, 1.87), 1.72 (1.21, 2.44) and 1.75 (1.073, 2.84), respectively (model 3). No significantly increased hazards were found for STEMI in women with AF at any time period. For NSTEMI, sHRs in women were non-significant in the first year, but were 1.60 (1.06, 2.41), 2.33 (1.52, 3.58) and 2.48 (1.40, 4.37) at 1 to <5 years, 5 to <10 years and ≥10 years, respectively (model 3).

In men with AF, sHR for MI was 2.27 (1.45, 3.54) in the first six months since AF diagnosis (model 3). After the first six months, no significantly increased hazards of MI were observed. For STEMI, HR was 2.75 (1.13, 6.69) in the first six months since AF diagnosis, as demonstrated in model 2. No time periods were associated with a significantly increased hazard of STEMI in the model with competing risks considered. The sHR of NSTEMI in men was 2.14 (1.15, 4.01) in the first six months since AF, and 2.27 (1.11, 4.62) at 0.5 to <1 year (model 3). After the first year, no significantly increased hazards of NSTEMI were observed in men with AF. Forest plots for the sHRs of MI, STEMI and NSTEMI by time since AF diagnosis (*Table 3*, model 3) are also presented in the Supplementary material online, *Figure S1*.

**Figure 2:**
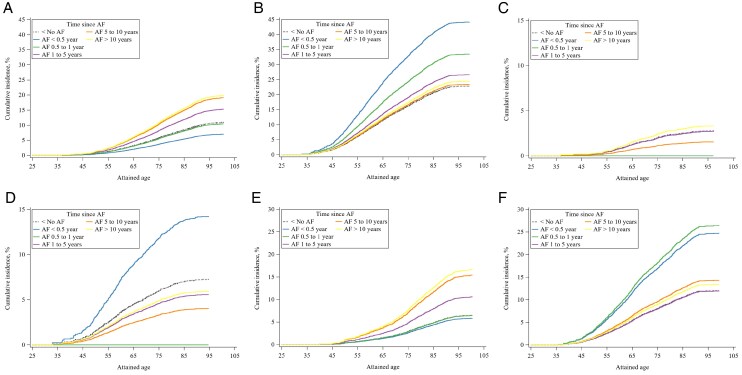
Cumulative incidence curves of myocardial infarction according to time since AF-diagnosis and by sex*. The Tromsø Study 1994–2021. (*Panel A*): MI in women. (*Panel B*): MI in men. (*Panel C*): STEMI in women. (*Panel D*): STEMI in men. Panel E: NSTEMI in women. Panel F: NSTEMI in men. AF; Atrial fibrillation. MI; Myocardial infarction. NSTEMI; non-ST-elevation myocardial infarction. STEMI; ST-elevation myocardial infarction. *Adjusted for age, body mass index, total cholesterol, high-density lipoprotein cholesterol, hypertension control (normotensive/controlled hypertension/uncontrolled hypertension/untreated hypertension), diabetes, hours of hard physical activity per week, current daily smoking and education level. Death, STEMI, NSTEMI, left bundle branch block, unclassified MI and fatal MI were treated as competing risks (death as a competing risk in analysis for total MI; death, NSTEMI, left bundle branch block, unclassified MI and fatal MI as competing risks in analysis for STEMI; death, STEMI, left bundle branch block, unclassified MI and fatal MI as competing risks in analysis for NSTEMI).

The adjusted cumulative incidence curves of MI, STEMI and NSTEMI by time since AF diagnosis are presented in *Figure 2*. These curves demonstrate a higher cumulative incidence of both MI, STEMI and NSTEMI in men than in women. Additionally, the temporal pattern described by the hazard ratios in the sections above is clearly reflected in the incidence curves.

### Sensitivity analysis

Adding cases with fatal MI, left bundle branch block and unclassified MI as STEMI-cases did not alter the results for women (see Supplementary material online, *Table S2*). Still, no significantly increased hazards of STEMI were observed at any time period. In men, however, the sHR of STEMI in the first six months since AF diagnosis, and by AF at any time, became statistically significant.

## Discussion

### Key findings

We found that AF is a risk factor for MI in both women and men. Specifically, AF is a risk factor for STEMI in men only, and for NSTEMI in both sexes. Furthermore, we found a time-dependent effect with different patterns in women and men. In women, we found a significantly increased hazard of NSTEMI *after* the first year since AF diagnosis. In men, we found a significantly increased hazard for STEMI only *during* the first six months since AF diagnosis, and for NSTEMI only *during* the first year since AF diagnosis, and then no significant HRs afterwards.

Our findings in women align with findings by Soliman *et al.* in the Atherosclerosis Risk In Communities-Study, where they found that AF was a risk factor for NSTEMI, but not STEMI.^4^ Our findings in men align with findings by Frederiksen *et al.* in the Framingham Study, where AF was a risk factor for both STEMI and NSTEMI.^5^ An important difference between the studies of Soliman *et al.*, Frederiksen *et al.,* and ours is that they all adjusted for sex, rather than presented sex-specific results, in their analyses of STEMI and NSTEMI after AF. While adjusting for sex accounts for its potential confounding effects, it assumes that the relationship between AF and MI risk is the same for both sexes. This approach may mask important sex differences in risk patterns. In sex-specific analysis, we were able to detect distinct risk profiles for STEMI and NSTEMI in women and men, which would likely have been missed if we had adjusted for sex instead. This highlights the importance of sex-specific analyses in studies where biological or clinical sex differences are plausible.

To our knowledge, no study has previously assessed the risk of MI, STEMI and NSTEMI by time since AF-diagnosis. However, a clustering of disease onset of AF and MI has been found, particularly during the first 30 days after each other.^7,8^ This aligns with our findings in men, where we found a significantly increased hazard of STEMI and NSTEMI after AF only during the first six and twelve months, respectively. Our findings in women are, however, contradictive—with no significantly increased hazard during the first year since AF, but with a significantly increased hazard of MI and NSTEMI after the first year since AF diagnosis, and onwards. The studies that have previously found a clustering of disease onset did not present sex-specific results.^7,8^ Again, our sex-specific analyses made it possible to discover the different temporal relationships between AF and MI, STEMI and NSTEMI in women and men, which are potentially hidden by adjusting for sex.

The observed sex difference in the temporal relationship between AF and MI is noteworthy. In men, we found that AF imposes an immediate, but short-lived, risk of MI. In women, however, AF needs to be prevalent for at least a year for it to become a significant, prolonged risk factor for MI. Women have, regardless of AF, been found to experience incident MI almost a decade later in life than men.^19–22^ Our study demonstrates that this is also true in AF-patients (see Supplementary material online, *Table S1*). A study from the Tromsø Study has previously demonstrated that the gender gap in incident MI, regardless of AF, cannot be explained by differences in classical cardiovascular risk factors or by female hormone levels.^20^

So why do women with AF experience MI later in life than men with AF? Women with AF have higher rates of stroke and thromboembolism and higher symptom burden than men with AF.^23–26^ Thus, AF is not more benign in women than in men. Moreover, there are significant sex differences in AF management. In women with AF, anticoagulation therapy is prescribed less frequently, atypical or asymptomatic AF is appropriately, less frequently treated with rhythm control, and referral to catheter ablation is less frequent and later, compared to in men with AF.^27–29^ Furthermore, women with AF are less likely to be assessed by a cardiologist, to get their low-density lipoprotein cholesterol tested and to receive statins.^30^ These disparities may explain parts of the sex-specific MI risk patterns observed in our study. The prolonged risk of NSTEMI in women may reflect a more prolonged exposure to risk factors such as endothelial damage, atherosclerosis, and microvascular dysfunction due to suboptimal anticoagulation, delayed rhythm control, and inadequate lipid management. In contrast, men’s higher short-term risk of STEMI and NSTEMI likely reflects the acute thromboembolic and hemodynamic effects of AF, combined with their higher prevalence of traditional cardiovascular risk factors. Recent evidence underscores the importance of systematic AF screening in improving clinical outcomes, especially thromboembolic events.^31^ Earlier detection of AF through screening could help address some of the disparities in MI risk patterns by enabling the timely initiation of anticoagulation therapy and other preventive measures. Screening strategies tailored to high-risk populations may be particularly beneficial in mitigating the prolonged risk of NSTEMI in women and the short-term risk of STEMI and NSTEMI in men.

Few studies have investigated sex differences in risk factor modification in patients with AF. Noubiap *et al.* offered risk factor management and participation in a tailored exercise program to 1415 patients with AF, and found a similar degree of weight loss and fitness gain in women and men.^32^ However, they observed that fitness had a greater benefit for total arrhythmia recurrence in women compared to men.^32^ Interestingly, Krijthe *et al.* found that in a population free of AF at baseline, unrecognized MI was associated with a twofold increased risk of AF in men, but not in women.^33^ As AF and (unrecognized) MI are likely to develop during the same time, it is plausible that the association also exists in the opposite direction and that AF is a risk factor for unrecognized MI in men.

### Strengths and limitations

The main strength of our study is the population-based cohort and longitudinal design. The Tromsø Study has a high participation, and individuals from both urban and rural areas are represented.^34^ The sample is representative of the general Norwegian population regarding the distribution of age and sex, but levels of education and physical activity are slightly higher.^35^ Furthermore, AF and MI are ascertained with high sensitivity. UNN is the only hospital in the area, and both out- and inpatient diagnoses of AF are included in the local registry. Both the Norwegian Myocardial Infarction Register and the local registry at UNN have been found to be highly complete and correct for MI diagnoses.^15^ Our analyses are comprehensive and demonstrate robustness as they remain significant or not significant after adjusting for cardiovascular risk factors and after taking competing risks into account. By using both Fine-Grey subdistribution hazard regression and Cox proportional hazard regression, our results are comparable with both other studies that have taken competing risk into account and studies that did not.

Our study has some limitations. We used unvalidated AF diagnosis from UNN from 2017 to 2021. For this period, it is possible that AF is overestimated. This could potentially affect our results in men, where we found an association with MI within the first year since AF diagnosis. In women, however, we find it unlikely that this affects our results, as the association between AF and MI was strongest 5 + years since AF diagnosis. Diagnostic criteria for MI evolved during the study period, particularly with the introduction of high-sensitivity troponin assays around the year 2000.^16^ This change likely resulted in a shift toward fewer STEMI and more NSTEMI diagnoses in later years.^36^ While we used validated MI diagnoses that adhered to consistent criteria within each time period, these changes may have influenced the observed patterns of STEMI and NSTEMI. Furthermore, our analysis of STEMI is underpowered, and a replication of the analysis in a larger population is warranted. Our study relied on clinic-based blood pressure (BP) measurements to classify hypertension status. While BP was measured using a standardized protocol with three measurements and the mean of the last two used to reduce variability, this approach may still be subject to misclassification compared to ambulatory or home BP monitoring. Such misclassification could weaken hypertension as a confounder in our models but is unlikely to have significantly influenced our conclusions. Additionally, there are some potential confounders missing. We have not included data on heart failure, kidney disease or AF treatment. Furthermore, all covariates adjusted for are measured at baseline only. The timeline from baseline to incident AF to incident MI can potentially be long, and it is likely that the levels of covariates change during this time, particularly after an AF diagnosis. Potential sex differences in risk factor management after AF diagnosis can possibly explain some of our findings.

### Implications

Our findings have implications for further research. We have demonstrated the importance of doing sex-specific analyses and encourage other researchers to do the same. Replications of the analysis in other populations are warranted to increase the statistical power and robustness of the findings. If reproducible in other populations, our findings may have clinical relevance and implications for practice and guidelines. We have identified an increased risk of NSTEMI in women 1–10+ years since AF diagnosis, which requires a longitudinal approach to risk factor management. In men, we identified an increased risk of STEMI within the first six months since AF diagnosis, and of NSTEMI within the first year since AF diagnosis. This calls for a more active approach and a closer follow-up of risk factor management in men, particularly during the first year since an AF diagnosis.

## Conclusion

We used data on 25 132 participants from a large, population-based cohort study to examine the risk of MI, STEMI and NSTEMI in women and men by time since first diagnosis of AF. We found that AF is a risk factor for MI in both women and men, but risk patterns differ. In women, we found no significant association between AF and STEMI, but an increased hazard of NSTEMI 1 to ≥10 years since AF diagnosis, with the highest hazard at ≥10 years, compared to women without AF. Men were at increased risk of STEMI within the first six months after AF diagnosis, and of NSTEMI within the first year since AF diagnosis, with no significant hazards observed beyond the first year. Our findings highlight the importance of doing sex-specific analyses and may have implications for different strategies for risk factor management in women and men with AF.

## Supplementary Material

qcag039_Supplementary_Data

## Data Availability

The data underlying this article were provided by the Tromsø Study by permission. Data from the Tromsø Study are available upon reasonable request and application. More information can be found at http://www.tromsoundersøkelsen.no.
