# Supplementary material for: Atrial fibrillation and risk of myocardial infarction by sex and time since diagnosis. The Tromsø Study 1994–2021
Source: Eur Heart J Qual Care Clin Outcomes. 2026 Apr 24;12(5):792–801. doi: 10.1093/ehjqcco/qcag039 (PMC13457917; doi:10.1093/ehjqcco/qcag039)
Supplement: Supplementary file 1 [file qcag039_supplementary_data.docx]

# Artikkel 3 – Supplementary material

**Supplementary table 1 – Descriptive characteristics by sex for participants with AF and MI^a^. The Tromsø Study 1994-2021.**

|  | **AF and not subsequent MI** | | **AF and subsequent MI** | | **MI and no AF** | |
| --- | --- | --- | --- | --- | --- | --- |
|  | Women | Men | Women | Men | Women | Men |
| n | 391 | 542 | 115 | 124 | 885 | 1487 |
| Age, years | 51.6 (9.4) | 47.8 (9.3) | 69.0 (9.5) | 60.8 (10.8) | 62.5 (13.0) | 54.3 (13.9) |
| Body mass index, kg/m^2^ | 26.1 (4.3) | 26.4 (3.3) | 26.9 (4.4) | 26.7 (3.8) | 26.5 (4.8) | 26.2 (3.5) |
| Total serum cholesterol, mmol/L | 6.31 (1.35) | 6.10 (1.08) | 7.07 (1.22) | 6.30 (1.18) | 7.09 (1.35) | 6.62 (1.18) |
| High density lipoprotein cholesterol, mmol/L | 1.67 (0.41) | 1.40 (0.36) | 1.68 (0.48) | 1.36 (0.36) | 1.57 (0.41) | 1.31 (0.34) |
| Hypertension control |  |  |  |  |  |  |
| Normotensive | 235 (60.1) | 305 (56.3) | 19 (16.5) | 32 (25.8) | 272 (30.7) | 613 (41.2) |
| Controlled hypertension | 6 (1.5) | 3 (0.6) | 4 (3.5) | 7 (5.6) | 17 (1.9) | 34 (2.3) |
| Uncontrolled hypertension | 29 (7.4) | 30 (5.5) | 29 (25.2) | 23 (18.5) | 141 (15.9) | 144 (9.7) |
| Untreated hypertension | 121 (30.9) | 204 (37.6) | 63 (54.8) | 62 (50.0) | 455 (51.4) | 696 (46.8) |
| History of diabetes | 4 (1.0) | 4 (0.7) | 6 (5.2) | 10 (8.1) | 49 (5.5) | 56 (3.8) |
| Hours of hard physical activity per week |  |  |  |  |  |  |
| None | 229 (58.6) | 162 (29.9) | 98 (85.2) | 71 (57.3) | 679 (76.7) | 717 (48.2) |
| Less than 1 | 80 (20.5) | 136 (25.1) | 10 (8.7) | 20 (16.1) | 93 (10.5) | 317 (21.3) |
| 1-2 | 56 (14.3) | 140 (25.8) | 5 (4.3) | 23 (18.5) | 78 (8.8) | 273 (18.4) |
| 3 or more | 26 (6.6) | 104 (19.2) | 2 (1.7) | 10 (8.1) | 35 (4.0) | 180 (12.1) |
| Daily smoker | 126 (32.2) | 155 (28.6) | 28 (24.3) | 31 (25.0) | 376 (42.5) | 655 (44.0) |
| Education |  |  |  |  |  |  |
| Primary education | 169 (43.2) | 148 (27.3) | 87 (75.7) | 53 (42.7) | 590 (66.7) | 649 (43.6) |
| Upper secondary education | 119 (30.4) | 186 (34.3) | 19 (16.5) | 40 (32.3) | 226 (25.5) | 517 (34.8) |
| University/college <4 years | 51 (13.0) | 106 (19.6) | 4 (3.5) | 18 (14.5) | 42 (4.7) | 193 (13.0) |
| University/college >=4 years | 52 (13.3) | 102 (18.8) | 5 (4.3) | 13 (10.5) | 27 (3.1) | 128 (8.6) |

a Numbers are given as mean (standard deviation) or number (percentage)

**Supplementary table 1 continued – Descriptive characteristics by sex for participants with AF and MI^a^. The Tromsø Study 1994-2021.**

|  | **AF and STEMI** | | **AF and NSTEMI** | |
| --- | --- | --- | --- | --- |
|  | Women | Men | Women | Men |
| n | 10 | 18 | 72 | 62 |
| Age, years | 70.0 (8.7) | 56.1 (10.8) | 67.8 (9.1) | 60.5 (12.0) |
| Body mass index, kg/m^2^ | 26.3 (3.9) | 26.4 (4.5) | 26.6 (3.9) | 26.8 (4.2) |
| Total serum cholesterol, mmol/L | 6.20 (1.28) | 5.90 (0.85) | 7.25 (1.23) | 6.30 (1.35) |
| High density lipoprotein cholesterol, mmol/L | 1.56 (0.53) | 1.29 (0.28) | 1.69 (0.47) | 1.38 (0.37) |
| Hypertension control |  |  |  |  |
| Normotensive | 1 (10.0) | 8 (44.4) | 14 (19.4) | 18 (29.0) |
| Controlled hypertension | 0 (0.0) | 1 (5.6) | 4 (5.6) | 4 (6.5) |
| Uncontrolled hypertension | 2 (20.0) | 1 (5.6) | 16 (22.2) | 9 (14.5) |
| Untreated hypertension | 7 (70.0) | 8 (44.4) | 38 (52.8) | 31 (50.0) |
| History of diabetes | 1 (10.0) | 2 (11.1) | 4 (5.6) | 6 (9.7) |
| Hours of hard physical activity per week |  |  |  |  |
| None | 7 (70.0) | 10 (55.6) | 61 (84.7) | 34 (54.8) |
| Less than 1 | 2 (20.0) | 5 (27.8) | 5 (6.9) | 12 (19.4) |
| 1-2 | 1 (10.0) | 2 (11.1) | 4 (5.6) | 12 (19.4) |
| 3 or more | 0 (0.0) | 1 (5.6) | 2 (2.8) | 4 (6.5) |
| Daily smoker | 2 (20.0) | 5 (27.8) | 17 (23.6) | 15 (24.2) |
| Education |  |  |  |  |
| Primary education | 7 (70.0) | 10 (55.6) | 53 (73.6) | 24 (38.7) |
| Upper secondary education | 2 (20.0) | 6 (33.3) | 15 (20.8) | 20 (32.3) |
| University/college <4 years | 1 (10.0) | 0 (0.0) | 2 (2.8) | 12 (19.4) |
| University/college >=4 years | 0 (0.0) | 2 (11.1) | 2 (2.8) | 6 (9.7) |

a Numbers are given as mean (standard deviation) or number (percentage)

**Supplementary table 1 continued – Descriptive characteristics by sex for participants with AF and MI^a^. The Tromsø Study 1994-2021.**

|  | **AF and left bundle branch block** | | **AF and fatal MI** | | **AF and unclassified MI** | |
| --- | --- | --- | --- | --- | --- | --- |
|  | Women | Men | Women | Men | Women | Men |
| n | 5 | 9 | 6 | 21 | 22 | 14 |
| Age, years | 71.80 (2.95) | 68.78 (6.78) | 61.50 (9.85) | 59.57 (6.25) | 74.00 (10.25) | 65.36 (9.75) |
| Body mass index, kg/m^2^ | 26.0 (10.6) | 26.7 (3.2) | 26.9 (4.6) | 27.1 (2.9) | 28.1 (4.7) | 26.2 (3.6) |
| Total serum cholesterol, mmol/L | 7.75 (1.42) | 6.51 (1.30) | 6.67 (0.94) | 6.60 (0.94) | 6.84 (0.96) | 6.21 (0.92) |
| High density lipoprotein cholesterol, mmol/L | 1.92 (0.69) | 1.24 (0.29) | 1.90 (0.67) | 1.32 (0.42) | 1.62 (0.35) | 1.46 (0.32) |
| Hypertension control |  |  |  |  |  |  |
| Normotensive | 0 (0.0) | 0 (0.0) | 3 (50.0) | 4 (19.0) | 1 (4.5) | 2 (14.3) |
| Controlled hypertension | 0 (0.0) | 0 (0.0) | 0 (0.0) | 6 (28.6) | 0 (0.0) | 2 (14.3) |
| Uncontrolled hypertension | 3 (60.0) | 4 (44.4) | 3 (50.0) | 11 (52.4) | 8 (36.4) | 3 (21.4) |
| Untreated hypertension | 2 (40.0) | 5 (55.6) | 6 (100.0) | 21 (100.0) | 13 (59.1) | 7 (50.0) |
| History of diabetes | 0 (0.0) | 1 (11.1) |  |  | 1 (4.5) | 1 (7.1) |
| Hours of hard physical activity per week |  |  |  |  |  |  |
| None | 4 (80.0) | 6 (66.7) | 5 (83.3) | 13 (61.9) | 1 (4.5) | 0 (0.0) |
| Less than 1 | 1 (20.0) | 0 (0.0) | 1 (16.7) | 3 (14.3) | 0 (0.0) | 4 (28.6) |
| 1-2 | 0 (0.0) | 1 (11.1) | 0 (0.0) | 4 (19.0) | 0 (0.0) | 2 (14.3) |
| 3 or more | 0 (0.0) | 2 (22.2) | 0 (0.0) | 1 (4.8) | 3 (13.6) | 6 (42.9) |
| Daily smoker | 3 (60.0) | 2 (22.2) | 3 (50.0) | 3 (14.3) | 1 (4.5) | 0 (0.0) |
| Education |  |  |  |  |  |  |
| Primary education | 4 (80.0) | 3 (33.3) | 4 (66.7) | 9 (42.9) | 19 (86.4) | 7 (50.0) |
| Upper secondary education | 1 (20.0) | 4 (44.4) | 0 (0.0) | 5 (23.8) | 1 (4.5) | 5 (35.7) |
| University/college <4 years | 0 (0.0) | 1 (11.1) | 0 (0.0) | 4 (19.0) | 1 (4.5) | 1 (7.1) |
| University/college >=4 years | 0 (0.0) | 1 (11.1) | 2 (33.3) | 3 (14.3) | 1 (4.5) | 1 (7.1) |

a Numbers are given as mean (standard deviation) or number (percentage)

**Supplementary Table 2 – Subdistribution hazard ratios for incident STEMI according to AF at any time or time since first diagnosis of AF – with cases of fatal MI, left bundle branch block and unclassified MI included as STEMI-cases*. The Tromsø Study 1994-2021.**

|  | **Women** | | | **Men** | | |
| --- | --- | --- | --- | --- | --- | --- |
|  |  | Subdistribution hazard ratio (95% confidence interval) | | | |  |
|  | **Model 1*** | **Model 2*** | **Model 3*** | **Model 1*** | **Model 2*** | **Model 3*** |
| No AF | **1 (Reference)** | **1 (Reference)** |  | **1 (Reference)** | **1 (Reference)** |  |
| Time since AF |  | | | | |  |
| 0 to <0.5 years | 0.47 (0.066, 3.39) | 0.41 (0.057, 2.94) | 0.45 (0.11, 1.83) | **2.37 (1.10, 5.067)** | **2.39 (1.18, 4.86)** | **2.22 (1.20, 4.09)** |
| 0.5 to <1 year | - | 0.55 (0.075, 4.00) | 0.94 (0.30, 2.95) | 0.48 (0.067, 3.44) | 0.42 (0.058, 3.00) | 0.89 (0.28, 2.79) |
| 1 to <5 years | 0.95 (0.46, 1.97) | 0.93 (0.47, 1.87) | 1.20 (0.75, 1.93) | 1.13 (0.65 1.97) | 1.46 (0.92, 2.31) | 1.48 (1.01, 2.18) |
| 5 to <10 years | 0.92 (0.37, 2.27) | 1.13 (0.52, 2.46) | 1.23 (0.69, 2.18) | 1.10 (0.55, 2.22) | 0.97 (0.48, 1.96) | 0.95 (0.52, 1.75) |
| ≥10 years | 0.68 (0.17, 2.77) | 0.93 (0.29, 2.94) | 1.07 (0.43, 2.63) | 1.53 (0.76, 3.082) | 1.54 (0.79, 2.98) | 1.18 (0.61, 2.29) |
| AF at any time | 0.78 (0.46, 1.32) | 0.89 (0.56, 1.44) | 1.13 (0.81, 1.58) | 1.27 (0.91, 1.78) | **1.37 (1.01, 1.87)** | **1.36 (1.04, 1.78)** |

Model 1: Fatal cases of MI included as STEMI-cases. Death, NSTEMI, left bundle branch block and unclassified MI treated as competing risks.
Model 2: Fatal cases of MI and cases with left bundle branch block included as STEMI-cases. Death, NSTEMI and unclassified MI treated as competing risks.
Model 3: Fatal cases of MI, cases with left bundle branch block and unclassified MI included as STEMI-cases. Death and NSTEMI treated as competing risks.
* Adjusted for age, body mass index, total cholesterol, high density lipoprotein cholesterol, hypertension control (normotensive/controlled hypertension/uncontrolled hypertension/untreated hypertension), diabetes, hours of hard physical activity per week, current daily smoking and education level as additional covariates.
AF; atrial fibrillation, MI; Myocardial infarction, STEMI; ST-segment elevation myocardial infarction, NSTEMI; non-ST segment elevation myocardial infarction.

**Supplementary Figure 1 – Subdistribution hazard ratios of MI, STEMI and NSTEMI according to time since AF diagnosis and by sex*. The Tromsø Study 1994-2021.**

MI	STEMI	NSTEMI

AF; Atrial fibrillation. MI; Myocardial infarction. NSTEMI; non-ST-elevation myocardial infarction. STEMI; ST-elevation myocardial infarction.
*Adjusted for age, body mass index, total cholesterol, high density lipoprotein cholesterol, hypertension control (normotensive/controlled hypertension/uncontrolled hypertension/untreated hypertension), diabetes, hours of hard physical activity per week, current daily smoking and education level. Death, STEMI, NSTEMI, left bundle branch block, unclassified MI and fatal MI treated as competing risks (death as competing risk in analysis for total MI; death, NSTEMI, left bundle branch block, unclassified MI and fatal MI as competing risk in analysis for STEMI; death, STEMI, left bundle branch block, unclassified MI and fatal MI as competing risk in analysis for NSTEMI)**.**
